# The structural basis for the interaction between the CAF1 nuclease and the NOT1 scaffold of the human CCR4–NOT deadenylase complex

**DOI:** 10.1093/nar/gks883

**Published:** 2012-09-12

**Authors:** Alain-Pierre Petit, Lara Wohlbold, Praveen Bawankar, Eric Huntzinger, Steffen Schmidt, Elisa Izaurralde, Oliver Weichenrieder

**Affiliations:** Department of Biochemistry, Max Planck Institute for Developmental Biology, Spemannstrasse 35, 72076 Tübingen, Germany

## Abstract

The CCR4–NOT complex plays a crucial role in post-transcriptional mRNA regulation in eukaryotic cells. It catalyzes the removal of mRNA poly(A) tails, thereby repressing translation and committing mRNAs to decay. The conserved core of the complex consists of a catalytic module comprising two deadenylases (CAF1/POP2 and CCR4a/b) and the NOT module, which contains at least NOT1, NOT2 and NOT3. NOT1 bridges the interaction between the two modules and therefore, acts as a scaffold protein for the assembly of the complex. Here, we present the crystal structures of the CAF1-binding domain of human NOT1 alone and in complex with CAF1. The NOT1 domain comprises five helical hairpins that adopt an MIF4G (middle portion of eIF4G) fold. This NOT1 MIF4G domain binds CAF1 through a pre-formed interface and leaves the CAF1 catalytic site fully accessible to RNA substrates. The conservation of critical structural and interface residues suggests that the NOT1 MIF4G domain adopts a similar fold and interacts with CAF1 in a similar manner in all eukaryotes. Our findings shed light on the assembly of the CCR4–NOT complex and provide the basis for dissecting the role of the NOT module in mRNA deadenylation.

## INTRODUCTION

The control of mRNA poly(A) tail length has a critical role in post-transcriptional gene regulation (1). Long poly(A) tails promote translation and counteract mRNA degradation. These effects are mediated by the cytoplasmic poly(A)-binding protein (PABP) (2). When bound to the poly(A) tail of mRNAs, PABP interacts with the eukaryotic initiation factor 4G (eIF4G), which binds to the 5′ cap structure through interactions with the cap-binding protein eIF4E (2). These interactions circularize the mRNA and stimulate translation by enhancing the recruitment of the small ribosomal subunit (2). mRNA circularization also protects the mRNA ends from exonucleolytic degradation (2). Conversely, shortening the mRNA poly(A) tail by deadenylases, represses translation and generally commits the mRNA to degradation in somatic cells (1–3). Indeed, the first step in bulk mRNA degradation is the removal of the mRNA poly(A) tail (4). Following deadenylation, the mRNA can be degraded from the 3′-end by the exosome (4). Alternatively, deadenylated mRNAs are decapped by the decapping enzyme DCP2 and subsequently degraded by the 5′–3′ exonuclease XRN1 (5).

Deadenylation therefore, plays a crucial role in the regulation of mRNA expression in eukaryotic cells. mRNA deadenylation is catalyzed by the consecutive action of two cytoplasmic deadenylase complexes (4). The PAN2–PAN3 complex is involved in an early phase of deadenylation and degrades the mRNA poly(A) tail in a distributive manner to ∼50–110 nucleotides, depending on the organism and the specific mRNA (6,7). The second, more rapid phase of deadenylation is catalyzed by the CCR4–NOT complex (7,8). The CCR4–NOT complex is sufficient for mRNA deadenylation in the absence of PAN2 (7–9). In addition to its role in mRNA deadenylation, the CCR4–NOT complex and associated proteins have been implicated in a broad range of biological processes, including transcription, ubiquitination and protein modification (3,10).

The conserved core of the human CCR4–NOT deadenylase complex consists of at least two modules (3,8,10–14). The first module is the catalytic module, which contains two subunits: CCR4a (or its paralog CCR4b), and CAF1 (or its paralog POP2) (11–13,15–17). The second module, termed the NOT module, consists minimally of NOT1, NOT2 and NOT3 (13–15,18). NOT1 acts as a scaffold protein and interacts with CAF1 (or POP2) and NOT2 (11–15,18). In turn, CAF1 (or POP2) recruits either one of the two CCR4 paralogs to the complex, and NOT2 recruits NOT3 (11–13,18).

The precise role of the NOT module in mRNA deadenylation is not completely understood. One known function of the NOT proteins is the stabilization of the complex (12,18–22). Accordingly, depletion of NOT1 abolishes deadenylation in *Drosophila melanogaster* cells and leads to destabilization of the additional subunits of the complex (21). A second role for the NOT module in mRNA deadenylation is to mediate the recruitment of the catalytic subunits to specific mRNA targets. Indeed, several sequence-specific RNA-binding proteins accelerate the deadenylation of their targets by recruiting the CCR4–NOT complex via interactions with either NOT1 or NOT3 (1,10). These RNA-binding proteins include Nanos, Bicaudal-C and Smaug, which regulate the temporal and spatial expression of maternal mRNAs during *D. melanogaster* oogenesis and embryogenesis (1,10). Similarly, proteins of the GW182 family, which are required for miRNA-mediated gene silencing in animal cells, interact directly with NOT1, thereby recruiting the CCR4–NOT complex to miRNA targets (23–25).

The interaction between NOT1 and CAF1/POP2 connects the NOT module to the catalytic module and thus is central to the assembly of the CCR4–NOT complex (3,10–12,15,18,19). Given the crucial role of this interaction, we have solved the crystal structures of the isolated CAF1-binding domain of human NOT1 (residues 1093–1317) at 2.90 Å resolution and its complex with CAF1 (i.e. CNOT7) at 2.70 Å resolution. The structures reveal that the CAF1-binding domain of NOT1 adopts an MIF4G fold; thus, this domain was termed the NOT1 MIF4G domain. MIF4G domains belong to the HEAT-repeat protein family and are found in proteins that are involved in post-transcriptional mRNA regulation (26). These proteins include eIF4G (27); the CBP80 subunit of the cap-binding complex (CBC) (28); PAIP1 (29) and UPF2 (30), an effector of the nonsense-mediated mRNA decay pathway. The MIF4G domain of NOT1 binds CAF1 opposite to its catalytic site, leaving the catalytic residues accessible to RNA substrates. Comparison of the structures of each partner free or in the complex indicate that the interfaces are preformed and that the interaction does not alter the protein folds. Mutagenesis of NOT1 and CAF1 shows the relevance of the interaction interface for both complex assembly and mRNA deadenylation.

## MATERIALS AND METHODS

### Coimmunoprecipitation assays in human and S2 cells

Plasmids expressing deadenylase subunits in human and *Dm* S2 cells were described previously (23). NOT1 and CAF1 mutants were generated by site-directed mutagenesis using the QuikChange Site-Directed Mutagenesis kit from Stratagene and the appropriate oligonucleotide sequences. Human HEK293T cells were grown in 10-cm dishes and transfected using Lipofectamine 2000 (Invitrogen). The transfection mixtures contained a total of 20 μg plasmid, including both HA-tagged and green fluorescent protein (GFP)-tagged proteins. After 2 days of transfection, cells were washed with PBS and lysed in radioimmunoprecipitation assay (RIPA) buffer [20 mM HEPES (pH 7.6), 150 mM NaCl, 2.5 mM MgCl_2_, 1% NP-40, 1% deoxycholate supplemented with protease inhibitors (Complete protease inhibitor mix, Roche)] for 15 min on ice. Cells were spun at 18 000*g* for 15 min at 4°C. Cleared cell lysates were treated with RNase A for 30 min and spun again at 18 000*g* for 15 min. A polyclonal anti-GFP-antibody was added to the lysates (dilution 1:500), and samples were incubated for 1 h at 4°C. Then, 50 μl of GammaBind G Sepharose (GE Healthcare) was added, and the mixtures were rotated for an additional hour at 4°C. Beads were washed three times with RIPA-wash buffer [20 mM HEPES (pH 7.6), 150 mM NaCl, 2.5 mM MgCl_2_, 0.1% NP-40] and once with PBS. Bound proteins were eluted with 100 µl of SDS–PAGE sample buffer and analyzed by western blotting.

Coimmunoprecipitations in S2 cells were performed as described previously (23). *Dm* S2 cells were grown in six well dishes, transfected using Effectene (Qiagen) transfection reagent and harvested 3 days after transfection. The transfections mixtures contained 3.5 µg of plasmid expressing GFP-tagged NOT1 and 0.5 µg of HA-tagged POP2 (wild-type or mutant). Alternatively, the transfection mixtures contained 0.25 µg of GFP-POP2 and 2 µg of HA-NOT1 (wild-type or mutant) or 2.5 µg of GFP-CCR4 and 0.5 µg of HA-POP2 (wild-type or mutant). A plasmid expressing GFP-F-Luc (5–10 ng) served as a negative control. HA and GFP-tagged proteins were detected using horseradish peroxidase (HRP)-conjugated monoclonal anti-HA (Roche 3F10; 1:5000) and anti-GFP antibodies (Roche 11814460001; 1:2000), respectively. All western blots were developed with the ECL western blotting detection system (GE Healthcare) as recommended by the manufacturer.

### *In vitro* pull-down assays

To express the *Hs*NOT1 middle domain (amino acids 1085–1605 and 1093–1317) in *Escherichia coli*, the corresponding cDNAs were respectively cloned into pETM41P and pnEA-NpM vectors [derived from the pETMCN series; (31)], resulting in N-terminal maltose binding protein (MBP) fusion proteins. cDNAs encoding *Hs* CAF1 (full-length or amino acids 9–260) and *Hs* POP2 were cloned into the pnEA-NpG vector (derived from the pETMCN series), resulting in an N-terminal glutathione S-transferase (GST)-tagged proteins. GST or a GST-tagged CAF1 or POP2 and MBP-tagged NOT1 fragments were expressed in BL21 star cells at 20°C overnight. Cells were lysed in 10 mM HEPES (pH 7.6), 300 mM NaCl, 1 mM DTT supplemented with lysozyme (1 mg/ml), DNaseI (5 µg/ml) and protease inhibitors. Cell lysates were sonicated and cleared by centrifugation. The cleared supernatants containing the respective binding partners were mixed to obtain an ∼1:1 ratio of the protein partners and incubated in binding buffer (10 mM HEPES (pH 7.6), 150 mM NaCl, 0.2% Triton X-100, 1 mM DTT and protease inhibitors) for 20 min at 4°C. Then, 50 µl (50% slurry) of Protino Glutathione Agarose 4B beads (Macherey Nagel) was added to each sample, and incubation was continued for another hour at 4°C with gentle rotation. Beads were washed three times with binding buffer. Bound proteins were eluted with 2× sample buffer and analyzed by SDS–PAGE.

### Protein expression and purification

The MIF4G domain of *Hs* NOT1 (ID: NP_057368.3; residues 1093–1317) was expressed in the *E. coli* BL21 Codon Plus RIL strain as a PresScission protease-cleavable His_6_ fusion. *Hs* CAF1 (ID: NP_037486.2) was expressed in the *E. coli* Rosetta2 strain as a PreScission protease-cleavable GST fusion. NOT1 and CAF1 were expressed in ZY autoinduction medium (32) and terrific broth medium, respectively, at 17°C overnight. NOT1 was purified by metal affinity chromatography (IMAC) using pre-charged Ni Sepharose columns (HisTrap HP) followed by gel filtration chromatography (Superdex 200, GE Healthcare). CAF1 was purified using a GSTrap column (GE Healthcare). A desalting step (HiTrap Desalting column) was added to remove glutathione prior to a second GST-purification after PreScission protease cleavage. The flow-through was finally purified by gel filtration chromatography (Superdex 200). The NOT1–CAF1 complex was obtained by incubating the purified NOT1 and CAF1 proteins (molar ratio 1.2:1) in 50 mM HEPES (pH 7.5), 200 mM NaCl, 1 mM TCEP [Tris(2-carboxyethyl)phosphine hydrochloride] and 10% glycerol at 4°C for 2 h. The complex was separated from the excess, free NOT1 by gel filtration chromatography on a Superdex 75 column.

### Crystallography

Before crystallization, the NOT1–CAF1 complex buffer was exchanged to 20 mM HEPES (pH 7.5), 200 mM NaCl, 1 mM TCEP and 10% glycerol using a HiTrap Desalting Column (GE Healthcare). The NOT1–CAF1 complex crystals were obtained by hanging-drop vapor diffusion method at 4°C within 10 days. To establish a hanging drop, 1μl of the protein complex solution (19 mg/ml) was mixed with 1 μl of the reservoir solution containing 0.1 M Tris–HCl (pH 8.2), 16% PEG 8000, 0.2 M MgCl_2_ and 0.2 M ammonium sulfate. Crystals were cryoprotected using the reservoir solution supplemented with 25% glycerol prior to flash freezing in liquid nitrogen. The NOT1 protein buffer was exchanged to 10 mM BTP (Bis–tris propane pH 7.2), 200 mM NaCl, 1 mM TCEP and 10% glycerol. NOT1 crystals were obtained using the same method by mixing 1μl of the protein sample [25 mg/ml, supplemented with V8 endoproteinase 1/5000 (w/w)] with 1μl of the reservoir solution containing 0.1 M BTP (pH 7.2), 1.6 M ammonium sulfate and 0.2 M LiCl at room temperature within 4 days. Crystals were flash-frozen in the reservoir solution mixed with 3.4 M sodium malonate at a 1:1 ratio.

### Data collection and structure determination

X-ray diffraction data were collected at 100 K at beamline PXII - X10SA (Swiss Light Source). The diffraction data of the NOT1–CAF1 complex were processed using XDS (33) and scaled using SCALA (34) from the CCP4 suite (35). The crystal diffracts to 2.70 Å and belongs to the space group P21. The initial phase information was obtained by molecular replacement using Phaser from the CCP4 suite and the coordinates of *Hs* CAF1 (PDB ID: 2D5R) as the search model. The initial densities were further improved by solvent flattening and histogram matching using RESOLVE (36), as implemented in the Phenix suite (37). The initial NOT1 model was built using AutoBuild from the Phenix suite and was further refined manually by iterative cycles of model building and refinement using COOT (38) and Refine (Phenix suite). A total of 211 water molecules were positioned in well defined positive (*F*_0_–*F*_C_) residual densities (cut-off of 3σ). Three chlorid atoms, eight magnesium atoms and three glycerol molecules were added to finalize the model (Supplementary Table S1). A similar method was used for the structure determination of free NOT1.

NOT1 diffraction data were processed in the space group C2221 and scaled to 2.90 Å. The initial phase was calculated using NOT1 coordinates from the NOT1–CAF1 complex as the search model. A total of 30 water molecules were added to finalize the model. Crystallographic model refinement and data collection statistics are presented in Supplementary Table S1. Representative figures of the crystal structure were created using PyMOL (http://www.pymol.org/).

### Luciferase assays in S2 cells

Transfection of S2 cells was performed in six-well dishes using Effectene transfection reagent (Qiagen). For the miRNA reporter experiments, the following plasmids were co-transfected: 0.1 µg of F-Luc-Nerfin-1 reporter plasmid, 0.4 µg pAc5.1-R-Luc (as transfection control) and 0.1 µg of pAc5.1 plasmid without insert (empty vector) or expressing miR-279 primary transcripts. To measure the mRNA half-life, transfected cells were treated with actinomycin D (5 µg/ml final concentration) 3 days after transfection and harvested at the indicated time points. RNA samples were analyzed as described previously (23).

## RESULTS AND DISCUSSION

### The mid region of NOT1 interacts with the catalytic module of the CCR4–NOT complex

**Figure 1.**
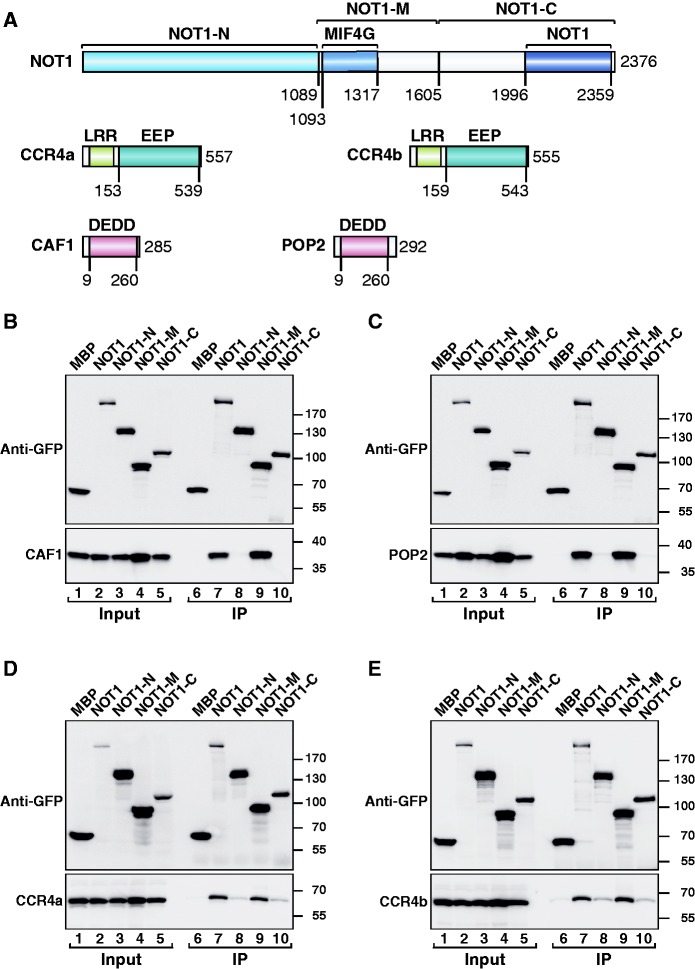
The middle region of human NOT1 interacts with the catalytic module of the CCR4–NOT complex. (**A**) Domain architecture of human NOT1, CCR4a/b and CAF1/POP2. NOT1 consists of an N-terminal (NOT1-N), middle (NOT1-M) and C-terminal (NOT1-C) region. The CAF1-binding domain crystallized in this study adopts an MIF4G fold and was termed the NOT1 MIF4G domain. The NOT1-C region contains a conserved NOT1 domain. CCR4a/b contain a LRR domain and an EEP-nuclease domain. CAF1 and POP2 consist of a single RNase D-like catalytic domain of the DEDD family. The numbers below the protein outline represent amino acid positions at fragment/domain boundaries. (**B–E**) GFP-tagged NOT1 (full-length or fragments) was co-expressed with HA-tagged catalytic subunits of the CCR4–NOT complex in HEK293T cells. NOT1 was immunoprecipitated from cell lysates using anti-GFP antibodies. GFP-tagged MBP served as a negative control. Inputs (1%) and immunoprecipitates (10% for the GFP-tagged proteins or 25% for HA-tagged proteins) were analyzed by western blotting.

To shed light on the assembly of the CCR4–NOT complex, we investigated the interactions between NOT1 and the subunits of the catalytic module (CAF1/POP2 and CCR4a/CCR4b) in human HEK293T cells. Sequence comparison and secondary structure predictions indicate that NOT1 consists of three regions that are mainly α-helical: the N-terminal (NOT1-N), middle (NOT1-M) and C-terminal (NOT1-C) regions (Figure 1A). The NOT1-C region contains a conserved NOT1 domain (Figure 1A). Human CCR4a and CCR4b are highly related proteins (78% identity) and consist of an N-terminal leucine-rich repeat (LRR) domain that interacts with CAF1 (or POP2) (11,15–17,39,40) and a C-terminal catalytic domain that belongs to the endonuclease–exonuclease–phosphatase (EEP) family of enzymes (41). CAF1 (i.e. CNOT7) and POP2 (i.e. CNOT8) are one-domain proteins that adopt an RNase D-like fold (42,43). They are members of the DEDDh subgroup of the DEDD family of nucleases and exhibit 74% identity. They both bind NOT1 in a mutually exclusive manner (13,16), thereby bridging the interaction of CCR4a (or CCR4b) with the NOT module (11–13).

To define the region in human NOT1 that mediates its interaction with the catalytic module of the CCR4–NOT complex, we performed coimmunoprecipitation assays in HEK293T cells using NOT1 deletion mutants. We observed that GFP-tagged NOT1 coimmunoprecipitated HA-tagged CAF1, POP2, CCR4a and CCR4b (Figure 1B–E, lane 7). These interactions were mediated by the NOT1-M fragment (Figure 1B–E, lane 9), as reported for the yeast proteins (12,18). Interestingly, we observed that POP2 accumulated at higher levels when co-expressed with NOT1 or the NOT1-M fragment, than when expressed alone or with NOT1 fragments with which it did not interact (Figure 1C, lanes 1–5), suggesting that POP2 is stabilized by NOT1 binding.

**Figure 2.**
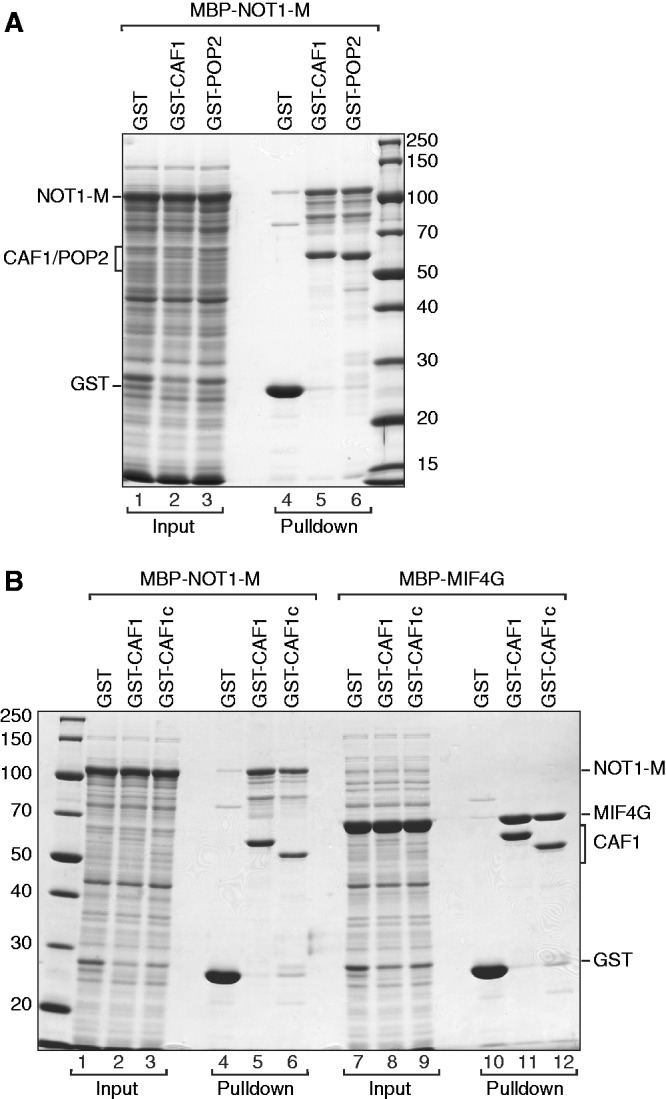
The interaction between NOT1 and CAF1/POP2 is direct. (**A**, **B**) Lysates from *E. coli* cells expressing GST or GST-fusions of CAF1 (full-length or the catalytic domain CAF1c) or POP2 were mixed with lysates from *E. coli* cells expressing MBP fusions of the NOT1-M fragment or the NOT1 MIF4G domain. GST-tagged proteins were pulled down using glutathione agarose beads. Input samples (1%) and bound fractions (100%) were analyzed by SDS–PAGE followed by coomassie blue staining.

To investigate whether the interaction between NOT1-M and CAF1 (or POP2) is direct, we performed pull-down assays with recombinant proteins expressed in *E. coli*. We found that both CAF1 and POP2 expressed with a GST-tag (Glutathione S-transferase) pulled down the isolated NOT1-M region, which was expressed with an MBP (maltose-binding protein) tag (Figure 2A, lanes 5 and 6), indicating that both CAF1 and POP2 interact directly with NOT1 and that this interaction does not require additional components of the complex.

Further inspection of the NOT1-M region indicated that it consists of a highly conserved N-terminal part (residues 1093–1317; Figure 1A) and a C-terminal region (1317–1605). Because the interaction of NOT1 with CAF1 (or POP2) is conserved, we speculated that this interaction is most likely mediated by the most conserved part of the NOT1-M region. Accordingly, we observed that the NOT1 fragment 1093–1317 interacted with CAF1 as efficiently as the entire NOT1-M region (Figure 2B, lane 11 versus lane 5). Furthermore, the folded core of the CAF1 nuclease (residues 9–260, CAF1c) was sufficient for the interaction (Figure 2B, lanes 6 and 12). We conclude that NOT1 interacts with the folded core of CAF1 via the N-terminal part of the middle region comprising residues 1093–1317. Interestingly, protein homology searches using HHpred (44) revealed that NOT1 fragment 1093–1317 exhibits sequence similarity to the MIF4G domains of PAIP1, eIF4G, CBP80 and UPF2; we therefore termed this fragment the NOT1 MIF4G domain (Figure 1A).

### Structural overview

**Figure 3.**
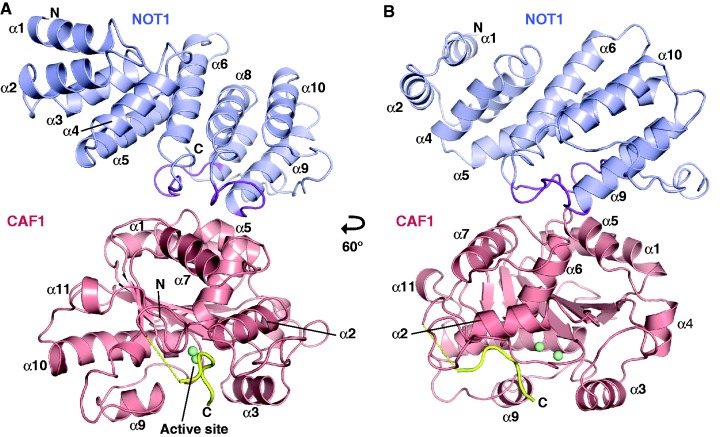
Structure of the NOT1 MIF4G domain in complex with CAF1. (**A, B**) Overview of the complex (cartoon representation). The CAF1-interacting parts of NOT1 loops L(α6–α7) and L(α8–α9) are colored purple. The two Mg^+2^ ions in the active site of CAF1 are drawn as green spheres. The C-terminal peptide of CAF1 (G274-E280) is colored yellow. Secondary structure elements are indicated. Views in panels (A) and (B) are related by a 60° rotation around the vertical axis.

To gain a more detailed understanding of the NOT1–CAF1 interaction, we solved the crystal structure of the MIF4G domain of human NOT1 (residues 1093–1317) alone and in complex with CAF1 (Figure 3). NOT1 and CAF1 proteins were expressed in *E. coli* and purified separately. The complex formed between the two proteins was purified by gel filtration (Supplementary Figure S1) and crystallized. The X-ray structure of the NOT1–CAF1 complex was determined by molecular replacement using a previously determined structure of human CAF1 as the search model [PDB ID code: 2D5R; (45)]. The structure was refined to 2.70 Å resolution with an *R*_free_ of 24% (Figure 3, and Supplementary Table S1 for phasing and refinement statistics). The crystals contain three complexes per asymmetric unit. These complexes are structurally very similar and superpose with root-mean-square deviation (RMSD) values ranging from 0.5 to 0.7 Å. The final structural model includes all residues of the NOT1 MIF4G domain and residues Q10–S266 and G274–E280 of CAF1 (chain B). The very N-terminal and some C-terminal residues of CAF1 are not visible in the electron density and are probably flexible or disordered.

The structure of the isolated MIF4G domain of NOT1 was solved by molecular replacement using NOT1 coordinates from the NOT1–CAF1 complex as the search model. The crystallographic asymmetric unit contains six monomers that superimpose to the NOT1 chains in the complex with RMSDs of <0.7 Å, indicating that the NOT1 structure is not altered upon binding to CAF1 (Supplementary Figure S2). The two structures have good stereochemistry, with ∼96.1% of the residues lying in the most favored regions of the Ramachandran plot (Supplementary Table S1).

### The CAF1-binding domain of NOT1 adopts an MIF4G fold

**Figure 4.**
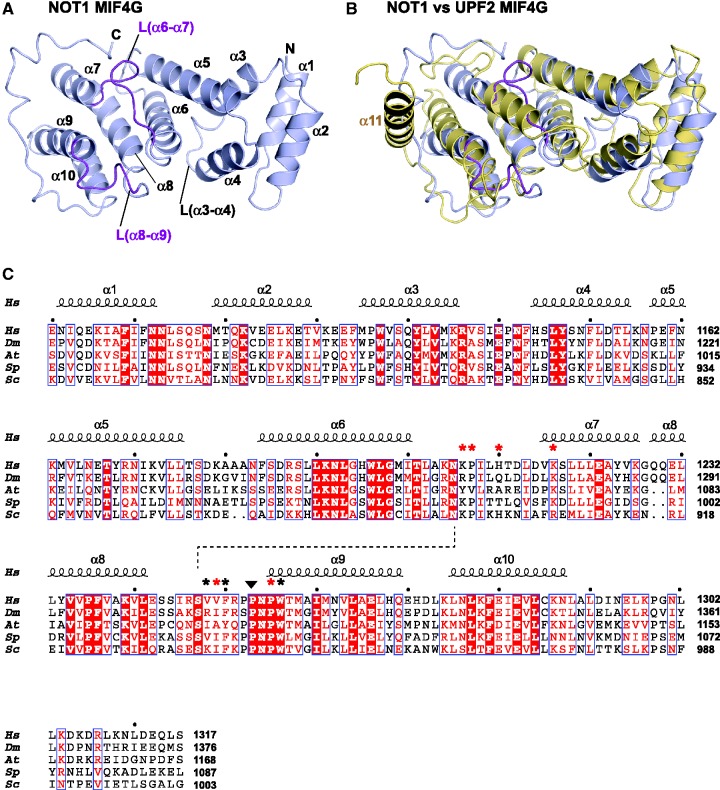
Structure of the NOT1 MIF4G domain. (**A**) Cartoon diagram of the NOT1 MIF4G domain. Secondary structure elements are indicated. The CAF1-interacting parts of loops L(α6–α7) and L(α8–α9) are colored purple. (**B**) Comparison of NOT1 and UPF2 (PDB ID: 1UW4) MIF4G domains. NOT1 and UPF2 are colored light blue and yellow, respectively. (**C**) Structure-based sequence alignment of NOT1 MIF4G domains from various species. Species are as follows: *Hs* (*Homo sapiens*), *Dm* (*D. melanogaster*), *At* (*Arabidopsis thaliana*)*, Sp* (*S. pombe*) and *Sc* (*S. cerevisiae*). Residues that are conserved in all aligned proteins are in red boxes, residues showing >70% similarity are printed in red, and the main residues involved in the interface are marked by asterisks (red if mutated in this study). The secondary structure elements derived from the structure of NOT1 are shown above the alignment and the conserved *cis*-proline is marked by a black triangle. The dashed line indicates a hydrogen bond between the invariant residues N1207 [L(α6–α7)] and S1249 [L(α8–α9)].

As predicted, the isolated MIF4G domain of NOT1 adopts a typical MIF4G fold composed of five HEAT-like antiparallel pairs of α-helices arranged into a right-handed solenoid (Figures 3 and 4A). The HEAT-like repeats are followed by a C-terminal extension that wraps around α-helices α9 and α7 (Figure 4A) in a structurally conserved manner, independent of crystal contacts (Supplementary Figure S2). A search of the structures deposited in the PDB using the program DALI (46) revealed that the NOT1 MIF4G domain is structurally most similar to the third MIF4G domain of human UPF2 [DALI *Z*-score 15.1; PDB ID: 1UW4; (30)], the MIF4G domain of CBP80 [DALI *Z-*score 14.4; PDB ID: 1H2U; (28)] and the middle domain of eIF4G [DALI *Z*-score 13.5; PDB ID: 2VSX; (47)]. A superposition of the NOT1 MIF4G domain with the third MIF4G domain of UPF2 yields an RMSD value of 2.8 Å, calculated from the positions of 190 alignable Cα atoms (Figure 4B), despite a very low sequence identity (<8%). The most notable difference between the MIF4G domains of NOT1 and UPF2 is that the C-terminal extension of NOT1 is replaced by an additional helix (α-helix 11) in UPF2 (Figure 4B) (30). Therefore, referring to the CAF1-binding domain of NOT1 as the NOT1 MIF4G domain is also justifiable based on its structural similarity to other MIF4G domains.

**Figure 5.**
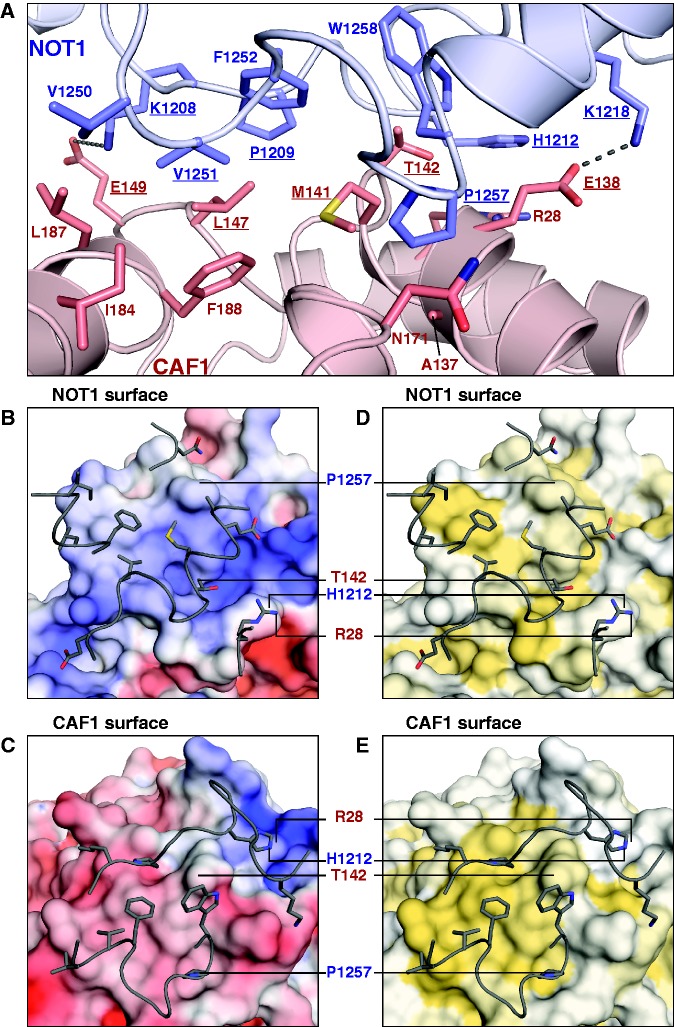
The NOT1–CAF1 interface. (**A**) Details of the interface (edge-on view, oriented as in Figure 3B). Selected side-chains are shown as sticks, with carbon atoms colored according to their respective main chains and oxygen and nitrogen in red and blue, respectively. Residues mutated in this study are underlined. (**B–E**) Interacting surfaces (plain view). To visualize the interacting surfaces, the binding partners as seen in (A) were rotated around the horizontal axis by 90°; upwards for the surface of NOT1 (B, D) and downwards for the surface of CAF1 (C, E). The interacting loops and side-chains of the respective binding partners (gray) are shown as tubes and sticks. Prominent side chains are labeled for orientation. (B, C) Electrostatic potential. Surfaces are colored according to the electrostatic potential, contoured from –5 kT/e (red) to +5 kT/e (blue). (D, E) Hydrophobicity. Surfaces are colored white to yellow with increasing hydrophobicity as described previously (48).

The amino acid sequence alignment of the NOT1 MIF4G domains from diverse species shows conservation of 41 residues that are distributed throughout the domain (Figure 4C). Many of these residues are part of the hydrophobic core of the domain or are involved in internal interactions between helices and thus, are required for the structural integrity of the domain. For instance, a cluster of highly conserved residues (L1192–T1203) in α-helix 6 plays a crucial role in maintaining the integrity of the protein fold by establishing interhelix interactions with helices α3, α5 and α8. However, some conserved residues are located in loops between helices. These residues are solvent-exposed and thus, likely to engage in protein–protein interactions (Figure 4C and Supplementary Figure S3). Particularly noteworthy are residues in the loops connecting α-helices α3 and α4, α6 and α7, α8 and α9. As described below, the residues in loops L(α6–α7) and L(α8–α9) are involved in binding CAF1 (Figure 3). Interestingly, these loops are mutually stabilized by a hydrogen bond between the invariant residues N1207 [L(α6–α7)] and S1249 [L(α8–α9)]. Furthermore, loop L(α8–α9) contains a well conserved PPNPW motif (Figure 4C), where N1256 constrains a backbone conformation that includes a *cis*-proline (P1255). As a consequence, one of the prolines (P1257) sticks like a knob into the hydrophobic surface of CAF1 (see below, Figure 5). Loop L(α6/α7) also contains a well conserved proline residue (P1209; Figure 4C), which, like P1257, is part of the hydrophobic core of the NOT1–CAF1 interface (Figure 5). The presence of proline residues in the NOT1 loops engaged by CAF1 likely allows for a close packing of the two protein partners and, together with the *cis*-conformation of P1255, provides an explanation for their conservation. Loop α3/α4 lies at the opposite side of the CAF1-binding site (Figure 4A and Supplementary Figure S3C); the conservation of residues in this loop suggests that it may provide a binding site for an as-yet unidentified protein partner or other parts of NOT1.

Importantly, the residues of the MIF4G domain of UPF2 that are involved in the UPF2–UPF3 interaction (30) are not conserved in the MIF4G domain of NOT1 (Supplementary Figure S4), indicating that NOT1 is unlikely to bind UPF3. Furthermore, the NOT1 MIF4G domain lacks the key residues that mediate binding to eIF4A in the MIF4G domains of eIF4G and PAIP1 (Supplementary Figure S4) (29,47). Thus, the sequence of the MIF4G domain of NOT1 has diverged from the sequences of other MIF4G domains, most likely to acquire the ability to interact with different partners.

### CAF1 binds the NOT1 MIF4G domain without undergoing major conformational changes

As expected, CAF1 adopts an RNase D-like fold with an open, twisted, mixed β-sheet of six β-strands surrounded by 10 α-helices (Figure 3). This fold is not altered by the binding of NOT1. Indeed, the structure of CAF1 bound to the MIF4G domain of NOT1 was solved by molecular replacement using the previously determined structure of CAF1 in complex with Tob as the search model (45). The model could be superposed with an RMSD of 0.4 Å to the structure of CAF1 bound to NOT1, indicating that the CAF1 fold remained unchanged in the complex with NOT1 (Supplementary Figure S5). Accordingly, the structure of human CAF1 is also highly similar to the structures of *Saccharomyces cerevisiae* and *Schizosaccharomyces pombe* Pop2 (43,49) with an overall Cα RMSD of 1.0 and 0.6 Å, respectively. Similarly, as mentioned above, the MIF4G domain of NOT1 does not undergo conformational changes upon binding to CAF1 (Supplementary Figure S2). Thus, both NOT1 and CAF1 interact with each other through preformed interaction interfaces.

Superposition of the CAF1 structures in complex with NOT1 and Tob revealed that these proteins can simultaneously bind CAF1 (Supplementary Figure S5). Tob belongs to a family of anti-proliferative proteins (the Tob/BTG family); these proteins are involved in cell cycle regulation in a variety of cells (45). Although the detailed mechanism of action of these proteins is not completely understood, Tob interacts with CAF1 (45,50–53). This observation led to the model that through its interaction with CAF1, Tob recruits the CCR4–NOT complex to mRNA targets and accelerates their deadenylation (3,45). In agreement with this model, Tob binding does not interfere with NOT1 binding (Supplementary Figure S5). Accordingly, Tob co-purifies with all core subunits of the CCR4–NOT complex indicating that it can indeed recruit the CCR4–NOT complex to mRNA targets (53). Furthermore, the CAF1 active site remains solvent-exposed in the Tob–CAF1 and NOT1–CAF1 complexes and in the putative trimeric Tob–CAF1–NOT1 complex (Supplementary Figure S5), consistent with the idea that these complexes are active and target bound mRNAs to deadenylation. Finally, Tob did not affect CAF1 nuclease activity *in vitro* (45). Nevertheless, it remains unclear how the Tob/BTG proteins influence CAF1 activity in the context of the CCR4–NOT complex, because these proteins were shown to both enhance and inhibit mRNA deadenylation (51–53).

### Details and conservation of the NOT1–CAF1 binding interface

The NOT1 MIF4G domain binds the convex surface of CAF1 (Figure 3). The buried interface is relatively small (712 Å^2^) and exhibits both shape and charge complementary (Figure 5A–E). The interaction is mediated primarily by residues from loops L(α6–α7) and L(α8–α9) in NOT1 and by residues from α-helices α1, α5, α6 and α7 in CAF1 (Figure 5A–E). Most of these residues participate in hydrophobic interactions, assisted by peripheral hydrogen bonds, π-stacking and electrostatic interactions. Most prominently, NOT1 residue P1257 (at the N-terminus of α-helix α9) functions as a central knob (Figure 5A); it inserts into the CAF1 surface between α-helices α5 and α6 and contacts A137, M141 (α-helix α5) and the C-beta atom of N171 (after the end of α-helix α6). Similarly, CAF1 residue M141 (α-helix α5) occupies a central position in the interface and is surrounded by NOT1 residues F1252, P1257 and W1258 (Figure 5A–E). T142 from the same α-helix also contacts W1258 and the C-beta atom of H1212, whereas CAF1 residue L147 [loop L(α5–β5)] bridges NOT1 residues P1209 [L(α6–α7)] and V1251 [L(α8–α9)]. Finally, V1251 is surrounded by I184, L187 and F188 (CAF1, α-helix α7), whereas the preceding V1250 only contacts I184 and L187.

The hydrophobic interactions are reinforced by a π-stacking interaction between H1212 [NOT1, L(α6-α7)] and R28 (CAF1, α-helix α1) and by peripheral salt bridges. In particular, residue K1218 (α-helix α7) is conserved among NOT1 homologs except in *S. cerevisiae*, where an arginine is found at the equivalent position (Figure 4C). K1218 forms a salt bridge (3.0 Å) with the highly conserved residue E138 (α-helix α5) of CAF1 (Figure 5A). At the opposite end of the interface, K1208 from NOT1 is spaced apart by 4.8 Å from residue E149 in CAF1 and hence could form another potential salt bridge (Figure 5A). However, as shown below and consistent with its peripheral position, mutations of the respective residues did not prevent complex formation in human or *D. melanogaster* cells.

Residues that form the interface are well conserved in NOT1 and CAF1 orthologs (Figure 4C and Supplementary Figure S6); however, some deviations are observed, suggesting that the relative contribution of charge-complementary and hydrophobic interactions to the affinity of the interaction may differ across species, as discussed below. Furthermore, human POP2 (74% identical to human CAF1) is very likely to interact with NOT1 in a similar way because all the interface residues are identical (Supplementary Figure S6).

### Orientation, architecture and accessibility of the CAF1 active site

As a consequence of the interaction between CAF1 and NOT1, the catalytic site of CAF1 is oriented toward the solvent without being obstructed by NOT1 (Figure 3). This finding illustrates how NOT1 acts as a scaffold to arrange the position of CAF1 as part of the CCR4–NOT complex while permitting free access for the RNA substrate and ensuring that CAF1 can remain catalytically active as a part of the complex.

**Figure 6.**
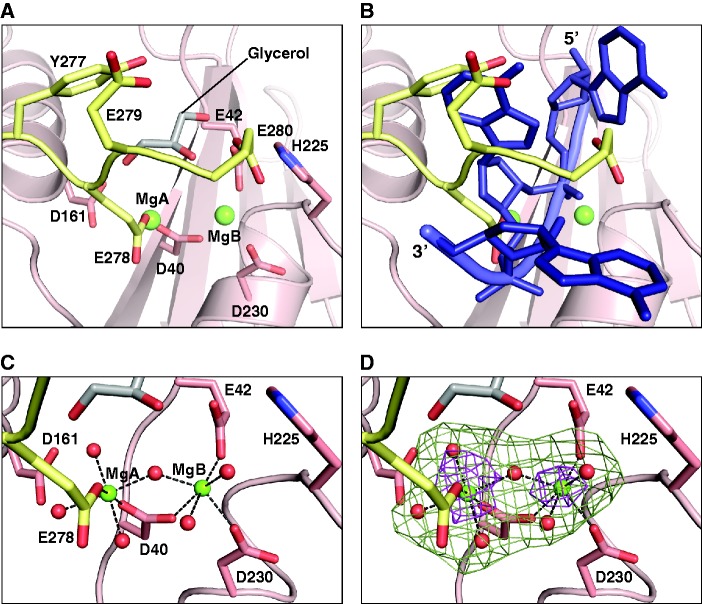
The CAF1 active site. (**A**) The C-terminal peptide of CAF1. In the crystal, the active site is blocked by a CAF1 C-terminal peptide (G274–E280, yellow) and a glycerol molecule (gray). (**B**) RNA binding model. The C-terminal peptide would clash with a single-stranded RNA substrate, modeled as found in the PARN nuclease (PDB ID: 2A1R) by a superposition of the active sites. (**C**) Enlarged view of the CAF1 catalytic site showing the coordination of Mg^+2^ ions. Hexagonal coordination by acidic side-chains and water molecules (red spheres) is indicated by dashed lines. Selected side chains are shown as sticks with oxygen in red and nitrogen in blue. (**D**) The Fo-Fc omit maps contoured at 9.0 (brown) and 3.0 (green) sigma reveal the location of the Mg^+2^ ions and of the coordinating water molecules, respectively.

CAF1 belongs to the RNase D superfamily and is characterized by a DEDDh-signature that is responsible for metal ion binding and enzymatic activity (Supplementary Figure S6) (43,49). Structure-based sequence comparisons of CAF1 orthologs shows that the key residues that form the core of the domain and the active site are strictly conserved, as reported previously (Supplementary Figure S6) (43,49). In particular, residues D40, E42, D161 and D230 of human CAF1 were predicted to bind to Mn^+2^/Mg^+2^ ions, whereas H225 was suggested to assist catalysis based on the two-metal ion mechanism (49). Due to the presence of 200 mM MgCl_2_ in the crystallization condition, we can now observe electron density for the catalytic metal ions in the human enzyme. Difference density in the active site was attributed to two hydrated magnesium ions (Mg_A_ and Mg_B_) and a glycerol molecule from the solvent (Figure 6A–D). The two magnesium ions have previously been observed in the structure of *S. pombe* Pop2p (PDB ID code: 2P51) (49), but their coordination in the present CAF1 structure shows some interesting differences. Similarly to the *S. pombe* structure, the more tightly coordinated metal ion in human CAF1 (Mg_B_) has inner sphere (direct) contacts to D230, E42 and D40 (Figure 6C). The central aspartate D40 also has an inner sphere contact to Mg_A_ and hence bridges the two metal ions (Figure 6C). In contrast to the *S. pombe* structure, however, Mg_A_ is additionally coordinated by residue E278, which establishes both inner sphere and outer sphere (water-mediated) contacts (Figure 6A and B). This coordination pattern results in a 0.7 Å shift of Mg_A_ compared with its counterpart in the *S. pombe* structure and to a replacement of the inner sphere contact between Mg_A_ and D161 by a water-mediated interaction (Figure 6C). Consequently, in our structure, the two metal ions are unusually close to each other (3.9 Å as compared with 4.6 Å in *S. pombe* Pop2p), similar to what has been described as an intermediate state in the reaction pathway of RNase H (54). In the context of RNase H, the rapprochement of Mg_A_ has been proposed to assist the geometry of the nucleophilic attack on the scissile phosphate and stabilize the high negative charge of the pentacovalent transition state (54).

Residue E278 belongs to the C-terminal tail of CAF1, which has not previously been observed in any CAF1 homolog deposited in the PDB. In the structure of the NOT1-CAF1 complex, the C-terminal CAF1 fragment encompassing residues G274–E280 is visible in the electron density (Figure 6A). This fragment includes three consecutive glutamates (E278–E280) and shields the active site of the enzyme (Figure 6A). Although the seven residues connecting the peptide to the nuclease domain cannot be traced, the arrangement of the molecules in the crystal indicates that the interaction is intramolecular. A superposition of the CAF1 catalytic site bound to the C-terminal peptide with the structures of the two closest homologs [human poly(A)-specific ribonuclease (PARN1) (RMSD 2.2 Å; PDB ID: 2A1R)] (55) and the 3′–5′ exonucleolytic active site of the Klenow fragment (RMSD 2.7 Å, PDB ID: 1QSL) (56) co-crystallized with a single-stranded RNA indicates that the peptide blocks access to the active site for potential RNA substrates (Figure 6B). This CAF1 C-terminal tail configuration suggests a mechanism for self-inhibition. However, the C-terminal sequences of the CAF1 orthologs are not highly conserved (Supplementary Figure S6), and the presence of three glutamic acid residues in the G274–E280 peptide together with 200 mM magnesium chloride from the crystallization condition may simply favor the observed interaction as a crystallization artifact. Nevertheless, the presence of a negatively charged peptide in the RNA-binding site suggests how CAF1 activity could be regulated in trans by related peptide sequences provided by other subunits of the complex or other regulatory proteins.

### Validation of the binding interface

**Figure 7.**
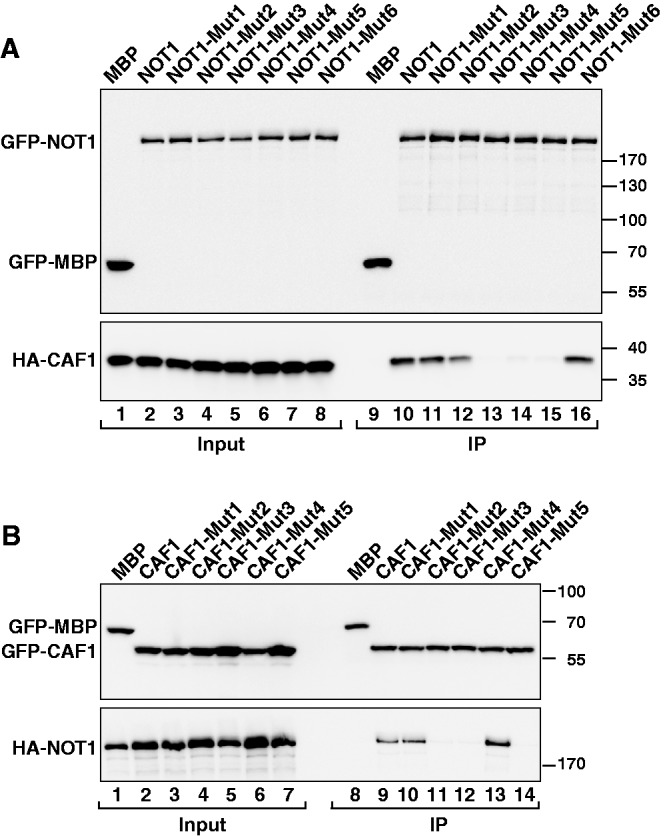
Mutagenesis of the NOT1–CAF1 interaction interface. (**A**) GFP-tagged NOT1 (wild-type or the indicated mutants) was coimmunoprecipitated with full-length HA-CAF1 and analyzed as described in Figure 1. (**B**) The interaction of GFP-tagged CAF1 (either wild-type or mutant) with NOT1 was analyzed as described in Figure 1.

To test whether the described interface between NOT1 and CAF1 is also relevant *in vivo*, we performed a mutational analysis and tested the NOT1–CAF1 interaction in coimmunoprecipitation assays. First, we generated mutations to disrupt two peripheral salt bridges and a central hydrophobic interaction*.* Specifically, we designed a triple K1208A-H1212A-K1218A mutation on the NOT1 MIF4G domain (NOT1-Mut1). Surprisingly, the triple mutations did not prevent the NOT1–CAF1 interaction (Figure 7A, NOT1-Mut1, lane 11). The interaction was reduced when residues K1208 and K1218 were substituted with glutamic acid and H1212 was substituted with tyrosine (Figure 7A, NOT1-Mut2, lane 12). The NOT1–CAF1 interaction was abolished when residue V1251 was substituted with arginine either in the context of the triple K1208A-H1212A-K1218A mutation or in isolation (Figure 7A, NOT1-Mut3 and NOT1-Mut-4, respectively). Conversely, alanine substitutions of CAF1 residues E138 and E149 (the salt bridge partners of K1218 and K1208) combined with the T142A mutation was ineffectual (Figure 7B, CAF1-Mut1, lane 10). In contrast, substitutions of CAF1 residues E138 and E149 with lysine combined with the T142Y mutation abolished complex formation (Figure 7B, CAF1-Mut2, lane 11).

Next, we analyzed the importance of the NOT1 proline residues for CAF1 binding by substituting P1209 and P1257 with tyrosine. These mutations abrogated CAF1 binding (Figure 7A, NOT1-Mut5, lane 15), most likely by interfering with the formation of close hydrophobic contacts. However, the P1257Y mutation alone was ineffectual (Figure 7A, NOT1-Mut6, lane 16). To further confirm the relevance of hydrophobic interactions for complex formation, we substituted CAF1 residues T142 with tyrosine and M141 and L147 with arginine (CAF1-Mut3). This triple mutation abolished NOT1 binding (Figure 7B, CAF1-Mut3, lane 12). Further analysis indicated that the T142Y mutation alone was ineffectual, whereas the M141R mutation was sufficient to abolish complex formation (Figure 7B, CAF1-Mut4 and CAF1-Mut5, respectively). Collectively, these results indicate that both hydrophobic and complementary charge interactions contribute to the formation of the NOT1–CAF1 complex in human cells. However, hydrophobic interactions are sufficient to mediate the interaction when residues involved in salt bridges are substituted with alanine.

### The NOT1–CAF1 complex interface is conserved in *D. melanogaster*

The human and *D. melanogaster* (*Dm*) proteins are conserved (67.1% and 61.4% sequence identity for the NOT1 MIF4G domain and CAF1/POP2, respectively). Thus, mutations based on the structure of the human NOT1–CAF1 complex can be easily mapped onto the *Dm* NOT1 and POP2 proteins (Figure 4C and Supplementary Figure S6). This similarity enabled us to investigate the effect of such mutations in the context of the full-length proteins in *D. melanogaster* Schneider cells (S2 cells).

**Figure 8.**
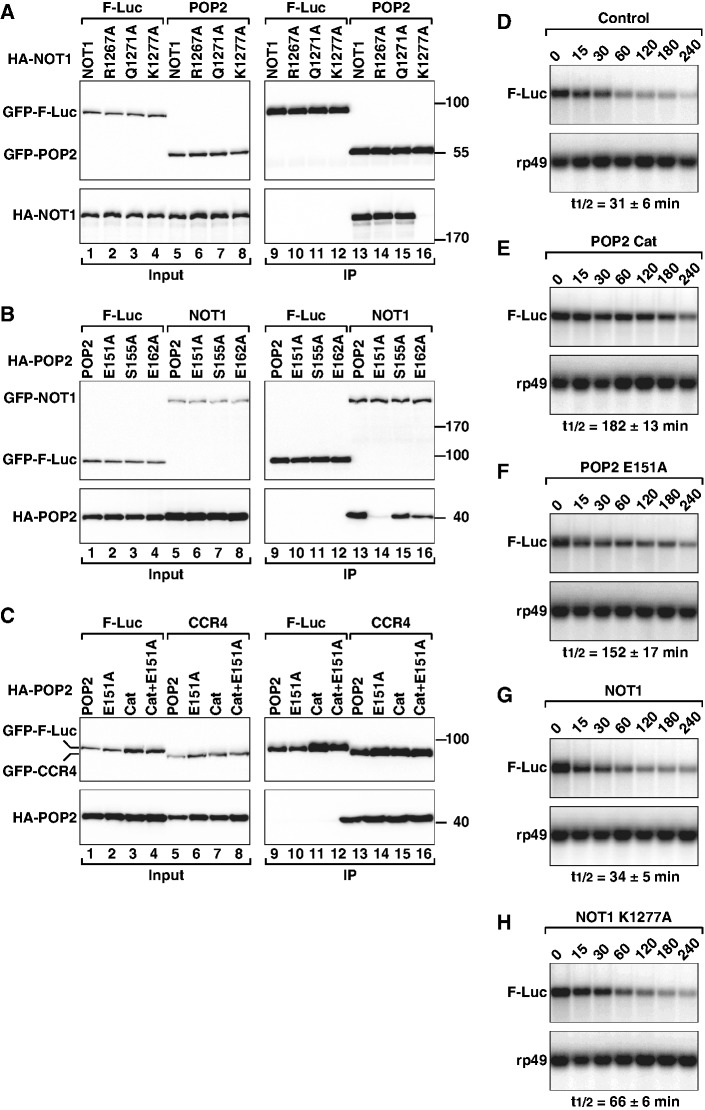
Mutagenesis of the *Dm* NOT1–CAF1 interaction interface. (**A**) S2 cells were transiently transfected with expression vectors encoding GFP-POP2 and HA-tagged NOT1 (wild-type or mutant). Cell lysates were immunoprecipitated using anti-GFP antibodies. GFP-F-Luc served as a negative control. (**B**) Interaction between GFP-tagged NOT1 and HA-POP2 (wild-type or mutant). (**C**) Interaction between GFP-tagged CCR4 and HA-POP2 (wild-type or mutant). POP2 catalytic inactive mutant (Cat) carries the following substitutions: D53A and E55A. In all panels, cell lysates were treated with RNase A, prior to immunoprecipitation. Inputs (1%) and immunoprecipitates (5% for the GFP-tagged proteins or 30% for HA-tagged proteins) were analyzed. (**D–H**) S2 cells were transfected with a mixture of three plasmids: one expressing the F-Luc-Nerfin-1 reporter, one expressing miR-279 primary transcript and one expressing *Renilla* luciferase (R-Luc). Plasmids for expression of POP2 or NOT1 (wild-type or mutant) were included in the transfection mixtures as indicated. The decay of F-Luc-Nerfin-1 mRNA was monitored following inhibition of transcription with actinomycin D. F-Luc-Nerfin-1 mRNA levels were normalized to rp49 mRNA and plotted against time. The mRNA half-lives (t_1/2_) calculated from the decay curves (not shown) are indicated below the panels. The mean values ± standard deviations from three independent experiments are shown.

To examine how *Dm* NOT1 mutations affect POP2-binding, GFP-tagged *Dm* POP2 was expressed in S2 cells, together with HA-tagged NOT1 wild-type or mutants. We then tested whether anti-GFP antibodies could coimmunoprecipitate HA-NOT1 from cell lysates. We found that alanine substitution of K1277 in the MIF4G domain of *Dm* NOT1 (corresponding to K1218 in human NOT1) was sufficient to abrogate the NOT1–POP2 interaction (Figure 8A, lane 16). In contrast, alanine substitutions of NOT1 residues Q1271 and R1267 (corresponding to H1212 and K1208 in human NOT1) were ineffectual (Figure 8A, lanes 14 and 15).

In POP2, we substituted E151 (corresponding to human E138), the salt bridge partner of NOT1 residue K1277, with alanine. This mutation also abolished binding (Figure 8B, lane 14). In contrast, alanine substitutions of POP2 residues S155 and E162 (corresponding to T142 and E149 in human CAF1) had only a minor effect (Figure 8B, lanes 15 and 16). The strong impact of the NOT1 K1277A and POP2 E151A mutations on complex formation is surprising because, as mentioned above, substitutions of the equivalent residues in the human proteins reduced but did not abolish the interaction. These results suggest that complementary charge interactions play a major role in complex formation for the *Dm* proteins, whereas hydrophobic interactions are dominant for the human proteins.

### The CAF1–NOT1 interaction is required for mRNA deadenylation

Previous studies showed that knockdown of the components of the CCR4–NOT1 complex or overexpression of a catalytically inactive CAF1 mutant strongly reduces miRNA-mediated mRNA deadenylation and decay both in *Dm* and human cells (23,24,57–60). Therefore, to study how the NOT1–CAF1 interaction impacts mRNA deadenylation, we examined the degradation of a previously characterized miRNA reporter consisting of the firefly luciferase open reading frame flanked by the 3′-UTR of the *Dm* gene Nerfin-1, which is silenced by miR-279. The rationale for using this reporter is that we showed in previous studies that its degradation depends on the CCR4–NOT complex (23,57,58).

In agreement with these previous studies, we observed that in the presence of miR-279, the half-life of F-Luc-Nerfin-1 mRNA was 31 min (Figure 8D). Overexpression of the catalytically inactive POP2 mutant (Cat, D53A+E55A) impaired miR-279-mediated decay of the F-Luc-Nerfin-1 mRNA, resulting in an mRNA half-life of 182 min (Figure 8E). Interestingly, a POP2 mutant that no longer interacted with NOT1 also inhibited miR-279-mediated degradation of the reporter, resulting in a 3.7-fold increase in the mRNA half-life (152 min; Figure 8F). This inhibitory effect was observed regardless of whether the POP2 mutant was in addition catalytically active or inert (Figure 8F and data not shown). These results suggest that a POP2 protein that does not interact with NOT1 inhibits mRNA degradation in a dominant-negative manner, most likely by sequestering CCR4 and preventing its incorporation into the endogenous complex. Indeed, the POP2 E151A mutant interacted with CCR4 independently of whether it was catalytically active (Figure 8C, lanes 14 and 16). Thus, a catalytic active POP2 protein that does not interact with NOT1 but interacts with CCR4 inhibits degradation, suggesting that the free CCR4–CAF1 module is not sufficient for degradation of miRNA targets. This finding is consistent with the observation that the CCR4–NOT complex is recruited to miRNA targets through interactions between GW182 proteins and NOT1 (23–25).

Next, we investigated the effect of overexpressing a NOT1 mutant (K1227A) that does not interact with POP2. We observed that in contrast to NOT1 wild-type, overexpression of this NOT1 mutant inhibited miR-279-induced degradation of the F-Luc-Nerfin-1 mRNA (Figure 8G and H), most likely by binding GW182 protein or other subunits of the CCR4–NOT complex, but failing to recruit the CCR4–POP2 module. Although the NOT1 mutant was less efficient than the POP2 mutants at inhibiting mRNA decay, the inhibitory effects of the two proteins cannot be directly compared because NOT1 is expressed at lower levels relative to POP2.

## CONCLUSIONS

Despite the importance of mRNA deadenylation in the regulation of gene expression, there is a paucity of structural information on the complexes that catalyze poly(A) tail degradation. Indeed, except for the catalytic domains of CAF1/POP2 and CCR4, until now, no structural information was available on the core subunits of the NOT module (e.g. NOT1–3). The structure of the NOT1–CAF1 complex presented here provides the first insight into how the catalytic and NOT1 modules of the CCR4–NOT complex interact to assemble a supramolecular complex. The structure confirms that NOT1 functions as a scaffold and demonstrates that NOT1 interacts with CAF1 without inducing conformational changes or interfering with the accessibility of the catalytic site. The structure together with structure-based mutational analysis of the interaction, provides a foundation for elucidating the role of the CCR4–NOT complex in the broad range of biological processes in which this complex has been implicated, including transcription and mRNA deadenylation.

## ACCESSION NUMBERS

The atomic coordinates of NOT1 and NOT1-CAF1 complex were deposited in the Protein Data Bank (PDB) under ID code 4GML and 4GMJ, respectively. 

## SUPPLEMENTARY DATA

Supplementary Data are available at NAR Online: Supplementary Table 1, Supplementary Figures 1–6 and Supplementary Reference [61].

## FUNDING

The Max Planck Society; the Deutsche Forschungsgemeinschaft [DFG, grant FOR855 to E.I.]; Gottfried Wilhelm Leibniz Program [awarded to E.I.]. Funding for open access charge: Max Planck Society.

*Conflict of interest statement*. None declared.

## Supplementary Material

Supplementary Data
