# Supplementary material for: The structural basis for the interaction between the CAF1 nuclease and the NOT1 scaffold of the human CCR4–NOT deadenylase complex
Source: Nucleic Acids Res. 2012 Sep 12;40(21):11058–72. doi: 10.1093/nar/gks883 (PMC3510486; doi:10.1093/nar/gks883)
Supplement: Supplementary Data [file supp_40_21_11058__index.html]

The structural basis for the interaction between the CAF1 nuclease and the NOT1 scaffold of the human CCR4–NOT deadenylase complex — The structural basis for the interaction between the CAF1 nuclease and the NOT1 scaffold of the human CCR4–NOT deadenylase complex — Supplementary Data 

# The structural basis for the interaction between the CAF1 nuclease and the NOT1 scaffold of the human CCR4–NOT deadenylase complex

## Supplementary Data

files

**Files in this Data Supplement:**

- Supplementary Data - pdf file
