# Supplementary material for: The structural basis for the interaction between the CAF1 nuclease and the NOT1 scaffold of the human CCR4–NOT deadenylase complex
Source: Nucleic Acids Res. 2012 Sep 12;40(21):11058–72. doi: 10.1093/nar/gks883 (PMC3510486; doi:10.1093/nar/gks883)
Supplement: Supplementary Data [file supp_gks883_nar-01823-v-2012-File010.pdf]

The structural basis for the interaction between CAF1 nuclease and the NOT1 scaffold of the human CCR4-NOT deadenylase complex

Alain-Pierre Petit, Lara Wohlbold, Praveen Bawankar, Eric Huntzinger, Steffen Schmidt, Elisa Izaurralde, Oliver Weichenrieder

Department of Biochemistry, Max Planck Institute for Developmental Biology, Spemannstrasse 35, 72076 Tübingen, Germany

Figure S1

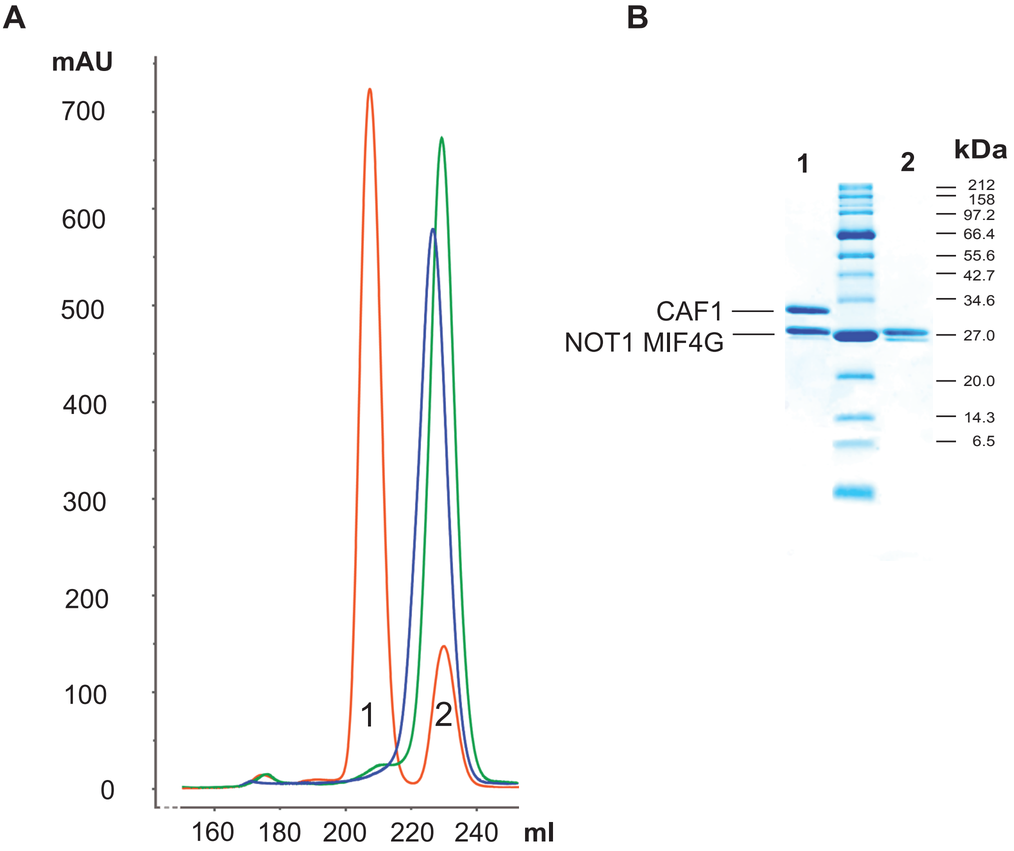

Figure S1. Gel filtration profiles of NOT1, CAF1, and the NOT1–CAF1 complex. (**A**) Analytical gel filtration chromatography was performed using an AKTA FPLC system (GE Healthcare). Proteins were loaded onto a Superdex 200 26/60 column (GE Healthcare) equilibrated with a buffer containing 50 mM HEPES (pH 7.5), 200 mM NaCl, 1 mM TCEP, 10% glycerol. Elution volumes measured for free NOT1 (green), free CAF1 (blue) and the complex (peak 1 - red curve) were 236, 225 and 210 ml, respectively. The complex was formed by incubation of purified NOT1 and CAF1 at a 1.2/1 molar ratio. The excess of NOT1 (peak 2 - red curve) was removed prior to the crystallization setup. (**B**) Gel filtration fractions were analyzed by SDS-PAGE (10%). A protein marker (Broad Range 2–212 kDa, NEB was included. Lanes 1 and 2 correspond to peaks 1 and 2 of the elution profile (red curve).

Figure S2

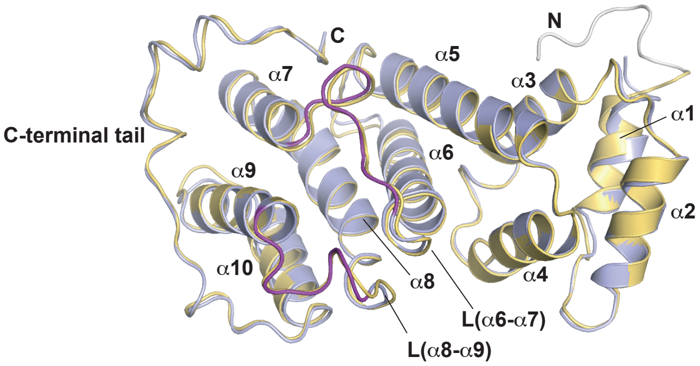

Figure S2. Superposition of the isolated NOT1 MIF4G domain (blue) with the one from the NOT1-CAF1 complex (yellow). The structure of NOT1 bound to CAF1 (yellow) contains additional N-terminal residues corresponding to the vector used for bacterial expression (white). The CAF1-interacting parts of loops L(α6-α7) and L(α8-α9) are colored purple. Secondary structure elements are indicated. Notably, the long C-terminal tail is structured identically in both cases, despite different crystal packing environments.

Figure S3

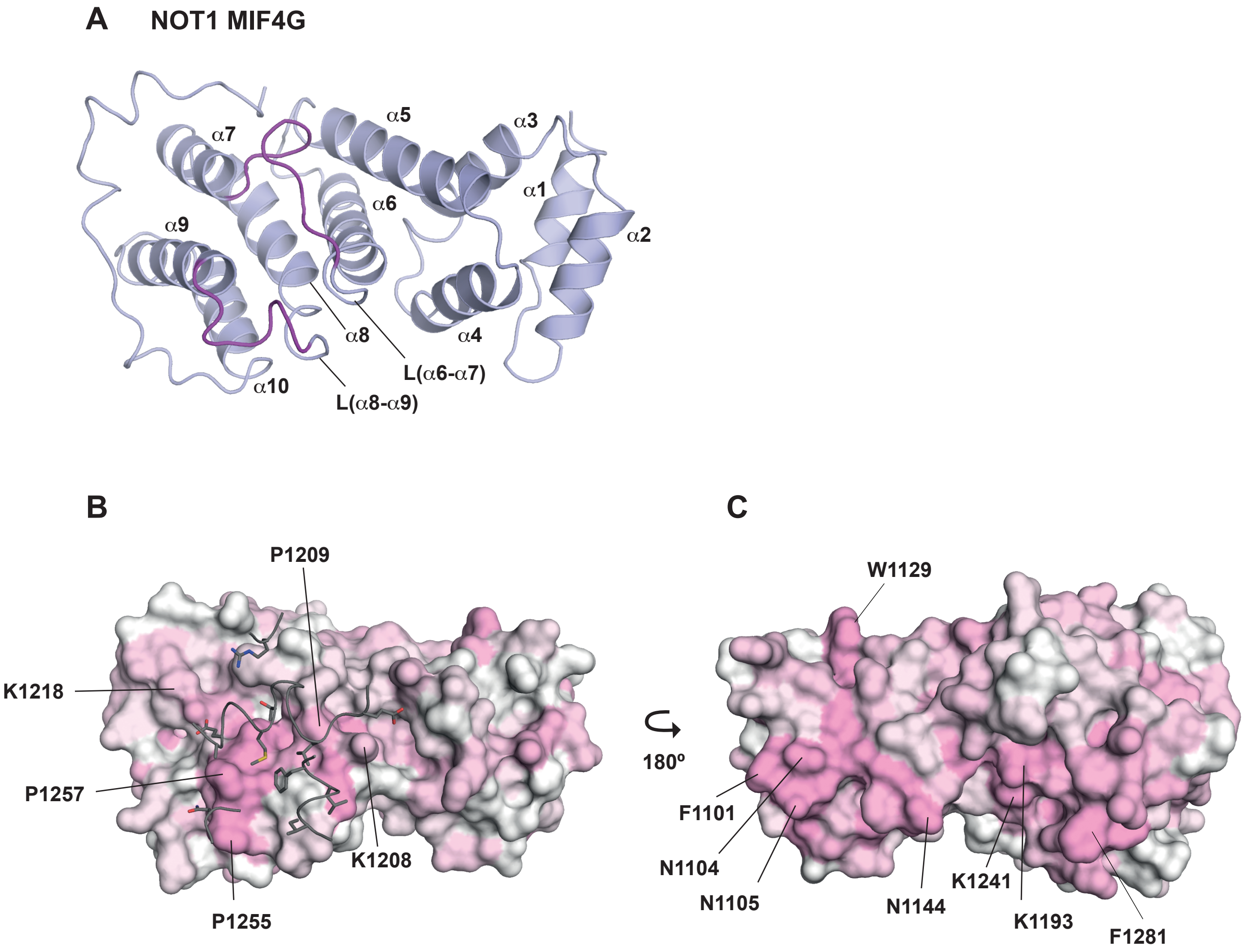

Figure S3. Conservation of the NOT1 MIF4G domain. (**A**) Cartoon representation of the NOT1 MIF4G domain. (**B,C**) Surface representation of the structure colored by sequence conservation comparing 4 species (Figure 4C). Color ramp by identity: magenta (100%) to white (0%). Panel B shows a plain view onto the surface involved in the interaction with CAF1. Selected residues are labeled. The interacting loops and side chains of CAF1 (gray) are shown as tubes and sticks, respectively. Conserved residues shown in panel (C) are not involved in CAF1-binding and may mediate additional protein-protein interactions. Views in panels (A) and (B) are in the same orientation. The view in panel (C) is related to (A) and (B) by a 180º rotation around the vertical axis.

Figure S4

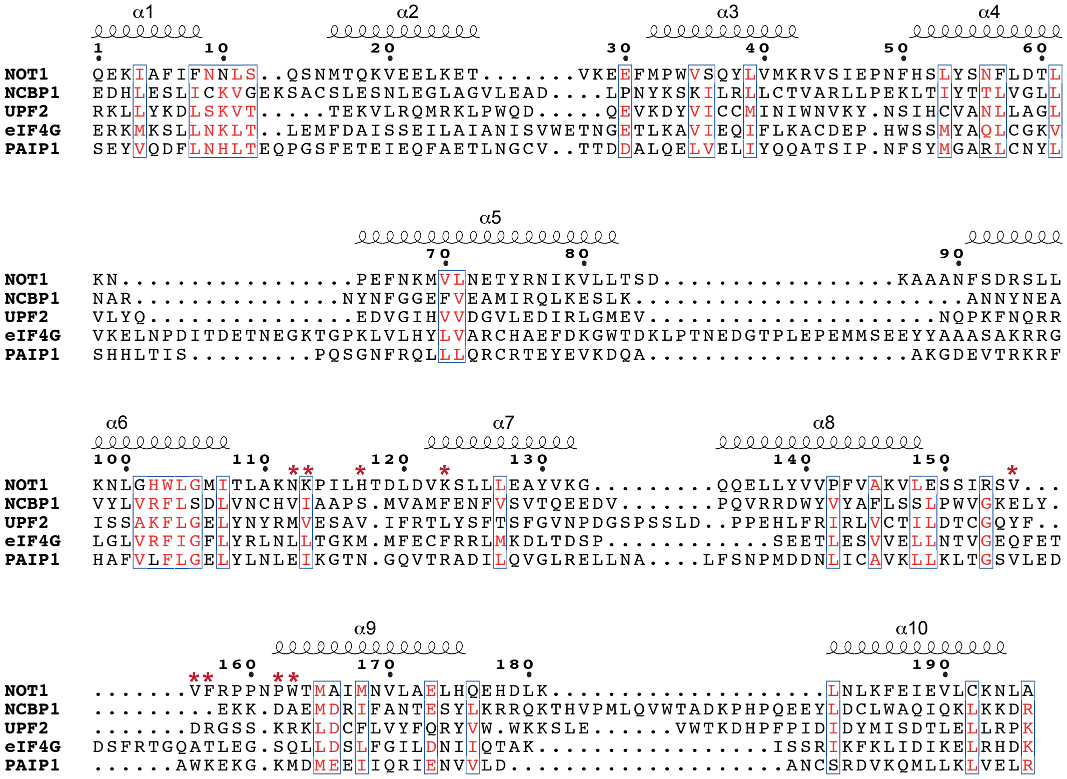

Figure S4. Multiple sequence alignment of *Hs* NOT1 with members of the MIF4G family with known structures. The alignment is based on the sequence family alignment ((26); PFAM PF02854) and a superposition of the relevant structures (human NCBP1 (PDB-id: 1h2u), human UPF2 (PDB-id: 1uw4), yeast eIF4G (PDB-id: 2vsx), and human PAIP1 (PDB-id: 3rk6)) using ESPript (61). The secondary structure of NOT1 is shown above, similar residues are highlighted in red (Risler, global score 0.7). The main residues involved in the NOT1-CAF1 interface are marked by red asterisks above the NOT1 sequence.

Figure S5

CAF1–NOT1 versus CAF1–Tob

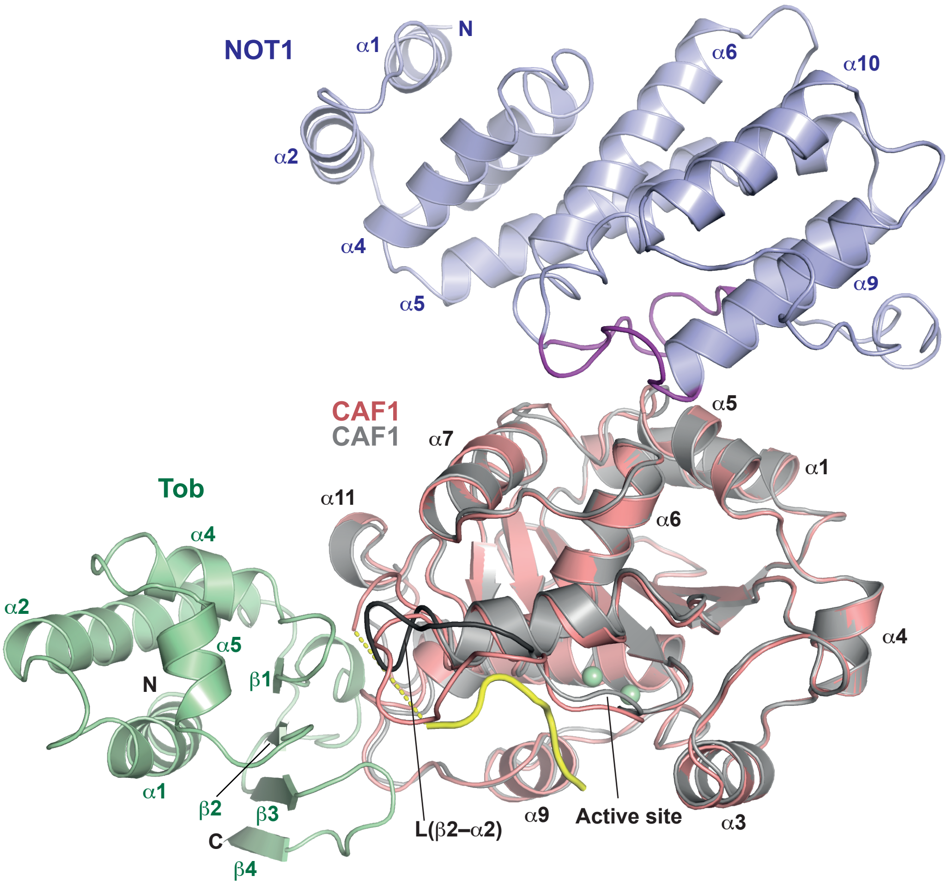

Figure S5. Superposition of the NOT1–CAF1 complex (view as in Figure 3B) with the Tob-CAF1 (PDB ID: 2D5R) complex. Tob is in green and does not interfere with NOT1 binding. CAF1 from the complex with Tob is in gray and is structurally highly similar with the exception of the missing C-terminal peptide and of the fold of loop L(β2–α2) (shown in black) that takes a different conformation.

Figure S6

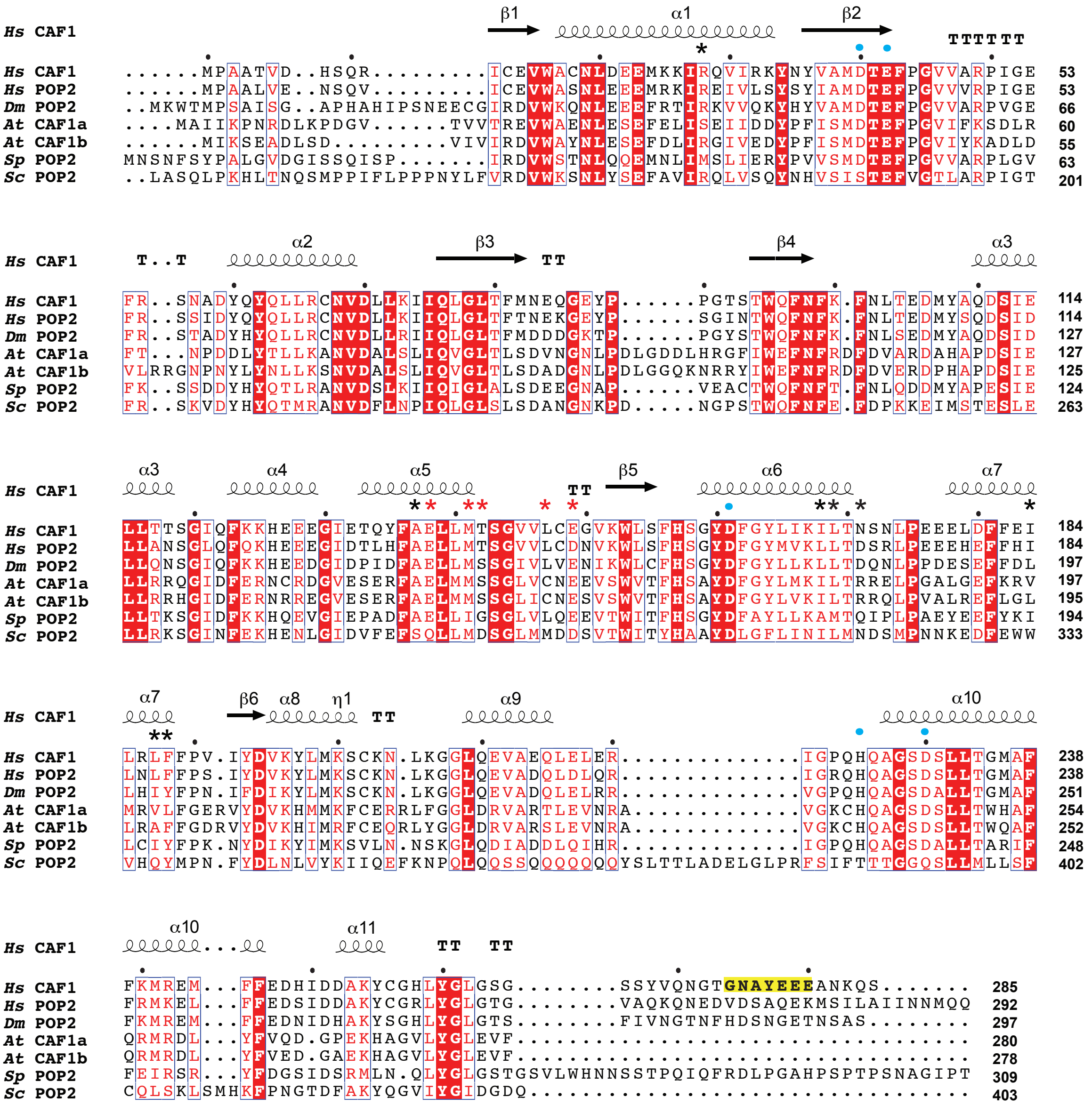

Figure S6. Structure-based sequence alignment of CAF1 with CAF1 proteins from other species. Species are as follows: *Hs* (*Homo sapiens*), *Dm* (*Drosophila melanogaster*), *At* (*Arabidopsis thaliana*), *Sp* (*Schizosaccharomyces pombe*), and *Sc* (*Saccharomyces cerevisiae*). Residues conserved in all aligned proteins are in red boxes, residues showing >70% similarity are printed in red, and the main residues involved in NOT1 binding are marked by asterisks. Asterisks are printed in red for residues mutated in this study. Catalytic residues are marked with cyan circles. Residues G274–E280 are marked by a yellow box. The secondary structure elements derived from the structure of CAF1 are shown above the alignment. The CAF1 secondary structure elements are labeled based on our structure. Relative to the CAF1 structure in complex with Tob, the 3_{10}-helix 1 is not visible and considered as an extension of the α-helix 1 in our structure (named α-helix 2 in 2D5R). The 3_{10}-helix 10 in 2D5R is labeled as η1 in our structure.

Table S1. Data collection and Refinement statistics

**Data collection**	**NOT1**	**NOT1-CAF1**
Space group	$C222_1$	$P2_1$
Cell dimensions		
A,B,C (Å)	88.27 133.82 331.84	82.47 101.74 142.20
α , β , γ (deg)	90.00 90.00 90.00	90.00 101.10 90.00
Wavelength (Å)	1.0	1.0
Resolutions (Å)	49.31-2.90 (3.06-2.90)[1]	82.21-2.70 (2.85-2.70)1
Unique reflections	44067 (6363)[1]	63214 (9227)[1]
Rmerge	0.089 (0.774)[1]	0.112 (0.676)[1]
I/ σ (I)	17.8 (3.2)[1]	11.0 (2.5)[1]
Completeness (%)	99.9 (100.0)[1]	99.6 (99.4)[1]
Redundancy	6.6 (6.9)[1]	3.6 (3.5)[1]
**Refinement**		
No. of atoms		
Protein	11102	11869*
Ligand	0	18
Ion	0	11
Water	30	211
R.m.s deviation		
Bond lengths (Å)	0.003	0.006
Bond angles (deg)	0.706	0.806
Rcryst	0.19	0.21
Rfree	0.23	0.24
Preferred (%)	96.10	97.80
Allowed (%)	3.80	2.20
Disallowed (%)	0.10	0.00

* Hydrogen atoms are not taken into account.
[1] Values in parentheses are for the outer shell
